# Thrombocytosis in a patient with acute promyelocytic leukemia during treatment with all‐*trans* retinoic acid and arsenic trioxide

**DOI:** 10.1002/ccr3.3978

**Published:** 2021-02-24

**Authors:** Maryam Habibi, Reza Manouchehri Ardekani, Hossein Motedayyen

**Affiliations:** ^1^ Autoimmune Diseases Research Center Kashan University of Medical Sciences Kashan Iran

**Keywords:** acute promyelocytic leukemia, all‐*trans* retinoic acid (ATRA), thrombocytosis

## Abstract

Thrombocytosis is an unusual side effect of all‐*trans* retinoic acid (ATRA) treatment that occurs in some patients with acute promyelocytic leukemia (APL).

## Graphical abstract

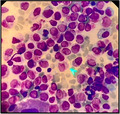

## 1 INTRODUCTION

Thrombocytosis, an uncommon side effect of all‐trans retinoic acid (ATRA) treatment, occurs in some patients with acute promyelocytic leukemia. Our case showed thrombocytosis on day 26 to day 32 of ATRA therapy and then started to decrease gradually without changing ATRA dosage. Thrombocytosis may associate with cytokines.

Acute promyelocytic leukemia (APL) results from a reciprocal chromosomal translocation t(15;17)(q24;q21) involving *retinoic acid receptor α (RARA)* and its fusion partners including the *promyelocytic leukemia (PML)* and *promyelocytic leukemia zinc finger (PLZF)*, which leads to the *PML‐RARA* chimeric gene formation.^1^, ^2^ This disorder is related to severe hemorrhagic syndromes and thrombotic problems, and abnormal accumulation of promyelocytes in the blood and bone marrow (BM).^3^, ^4^ Unlike other forms of AML, APL is well treated by all‐*trans* retinoic acid (ATRA; also known as tretinoin) therapy, as criteria to distinguish this disorder from other forms of AML.^4^

In most cases, ATRA therapy is well tolerated and its toxicity is modest. Hyperleukocytosis and the retinoic acid syndrome are two known complications. However, other side effects have been reported for APL including cheilosis, hypertriglyceridemia, headache, bone pain, pseudotumor cerebri, skin dryness, and mucous membranes.^5^ They are typically short term and simply controlled by other therapeutic approaches.^3^, ^6^

In this report, we reported a relatively uncommon side effect observed in a patient with APL during ATRA treatment.

## 2 CASE HISTORY

**FIGURE 1:**
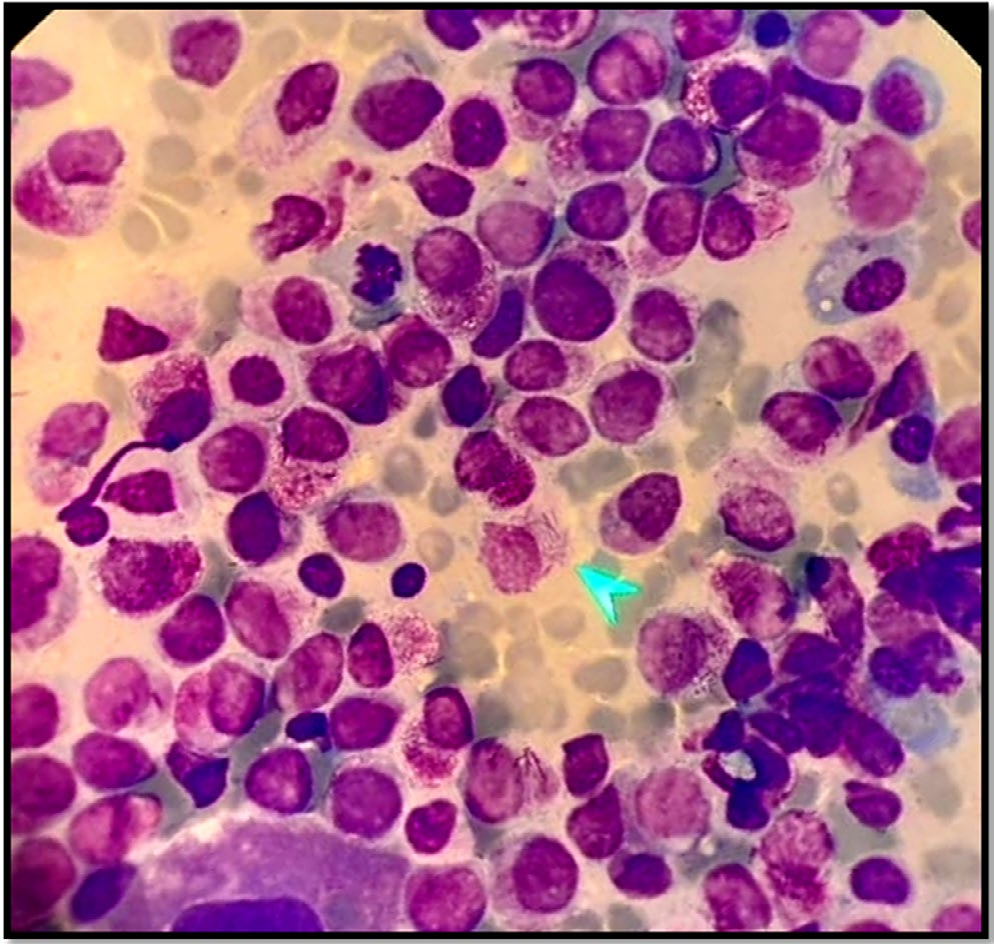
BM examination in a newly diagnosed APL patient (100×). BM examination revealed abnormal accumulations of promyelocytes (faggot cells) containing multiple Auer rods in the cytoplasm (arrow)

The patient was a 28‐year‐old woman from Afghanistan without familial or personal history of blood problems or malignancies and history of any specific illness or medication. The informed consent was obtained from patient prior to study initiation and all experimental protocols were approved by the Ethics Committee of Kashan University of Medical Sciences. At the time of termination of pregnancy in Shahid Beheshti hospital, Kashan, Iran, she had pancytopenia (Table 1). The patient was investigated to find the cause of pancytopenia. After termination of pregnancy, BM aspiration was carried out and its examination revealed abnormal accumulation of abnormal promyelocytic blasts. Promyelocytes included approximately 30% of total BM cells (Figure 1). Real‐time polymerase chain reaction (RT‐PCR) showed a PML‐RARA fusion transcript. Low‐risk APL (AML M3) was diagnosed according to the Sanz score.^7^, ^8^ Patient was initially treated with oral ATRA (45 mg/m^2^/day) and intravenous arsenic trioxide (ATO, 0.15 mg/kg/day) until complete remission achievement. On day 26 of ATRA therapy, the patient complained of blurred vision due to retinal bleeding and had decreased consciousness, headache, and seizure. Magnetic resonance imaging (MRI) result showed intracerebral parenchymal bleeding in the frontal lobe. The patient had no history of any specific trauma or head injury. After seizure control, the patient was treated with supportive therapies such as intravenous levetiracetam (500 mg BD) and intravenous dexamethasone (8 mg/12 hours). According to the neurosurgeon consultation, the patient did not need surgery. Furthermore, some laboratory tests were employed to exclude coagulation problems and find the cause of the bleeding. Similar to the results at the initial diagnosis, no thrombotic and hemorrhagic problems were observed (Table 2).

**TABLE 1:** Laboratory features of patient at the initial diagnosis

Laboratory parameter	Value	Normal range
WBC	1.2 × 10^9^/L (PMN 35%, lymphocyte 65%)	3.5‐12 × 10^9^/L
Hemoglobin (Hb)	8 g/dL	13.0‐17.0 g/L
Platelet counts	60 × 10^9^/L	150‐450 × 10^9^/L
Prothrombin time (PT)	13 s	9.4‐12.5 s
Activated partial thromboplastin time (aPTT)	25 s	25.1‐36.5 s
The international normalized ratio (INR)	1.16	1‐1.13
Fibrinogen	338 mg/dL	200‐400 mg/dL
Fibrin degradation products (FDPs)	3 µg/mL	Up to 5
D‐dimer	0.4 µg/mL	<0.5 µg/mL

**TABLE 2:** Laboratory findings of patient on day 26 of treatment

Laboratory parameter	Value	Normal range
WBC	3.6 × 10^9^/L (PMN 35%, lymphocyte 65%)	3.5‐12 × 10^9^/L
Hemoglobin (Hb)	7.8 g/dL	13.0‐17.0 g/L
Platelet counts	590 × 10^9^/L	150‐450 × 10^9^/L
Prothrombin time (PT)	13 s	9.4‐12.5 s
Activated partial thromboplastin time (aPTT)	25 s	25.1‐36.5 s
The international normalized ratio (INR)	1.16	1‐1.13
Fibrinogen	386 mg/dL	200‐400 mg/dL
Fibrin degradation products (FDPs)	5 µg/mL	Up to 5
D‐dimer	0.3 µg/mL	<0.5 µg/mL

**FIGURE 2:**
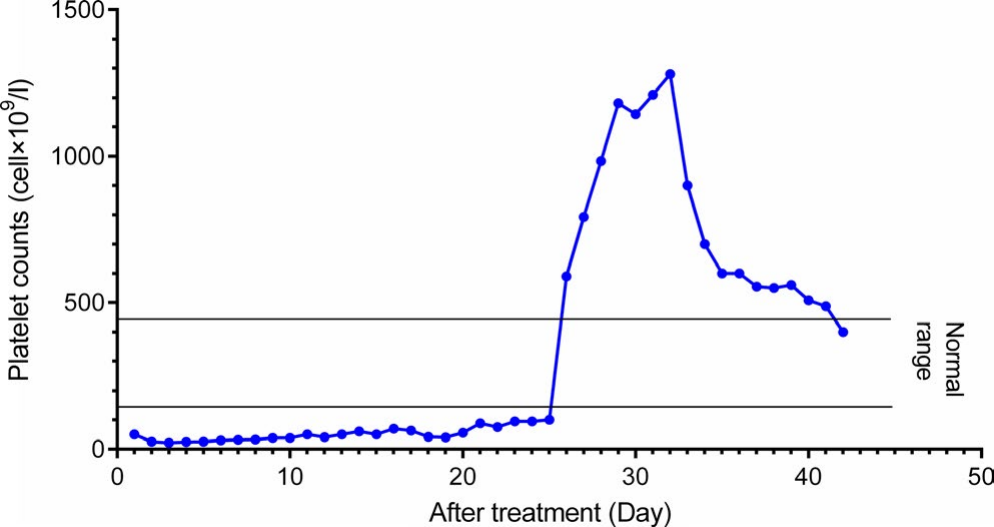
Platelet count curve from day 1 to day 42 of treatment with ATRA. Laboratory blood tests revealed the elevated numbers of platelets on day 26 to day 32 of treatment which this increase started to recover spontaneously on day 32 of treatment

**FIGURE 3:**
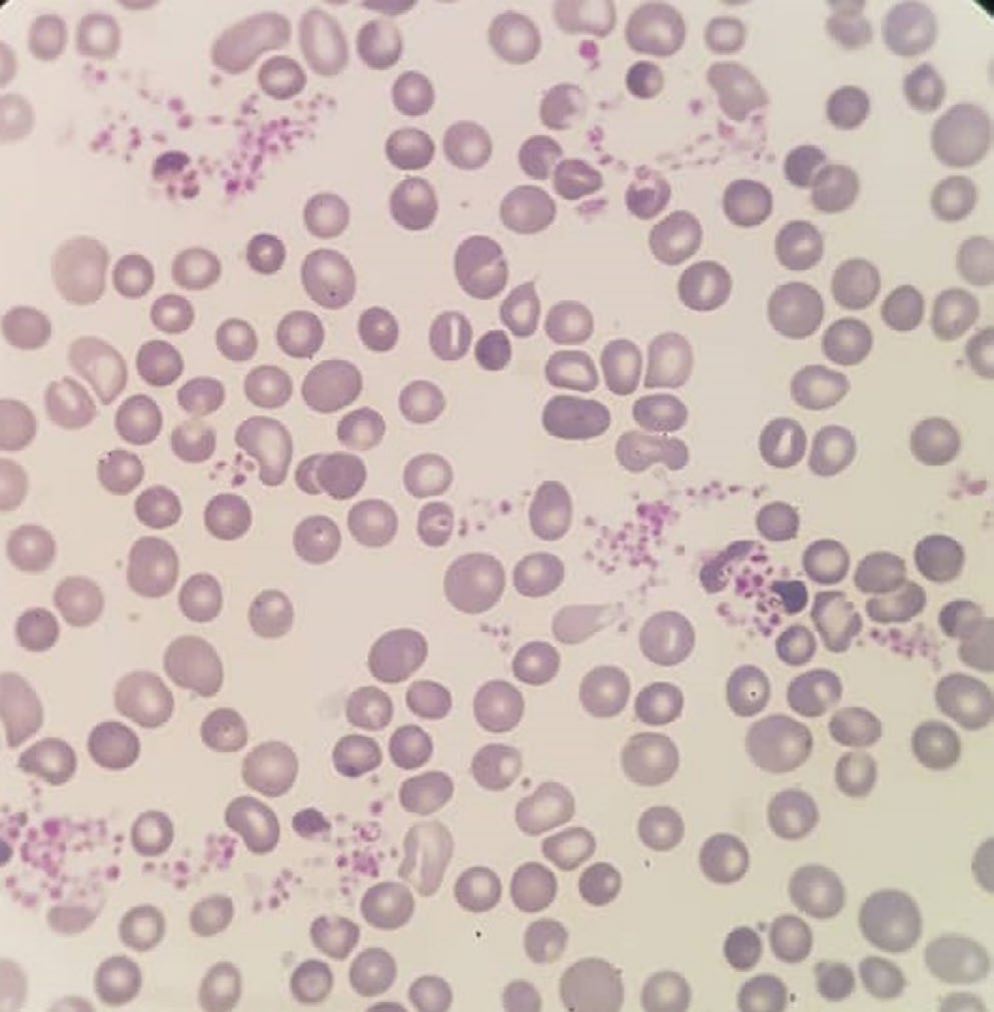
Peripheral blood smear of our patient (100×). Peripheral blood smear revealed a thrombocytosis and slight anemia

**FIGURE 4:**
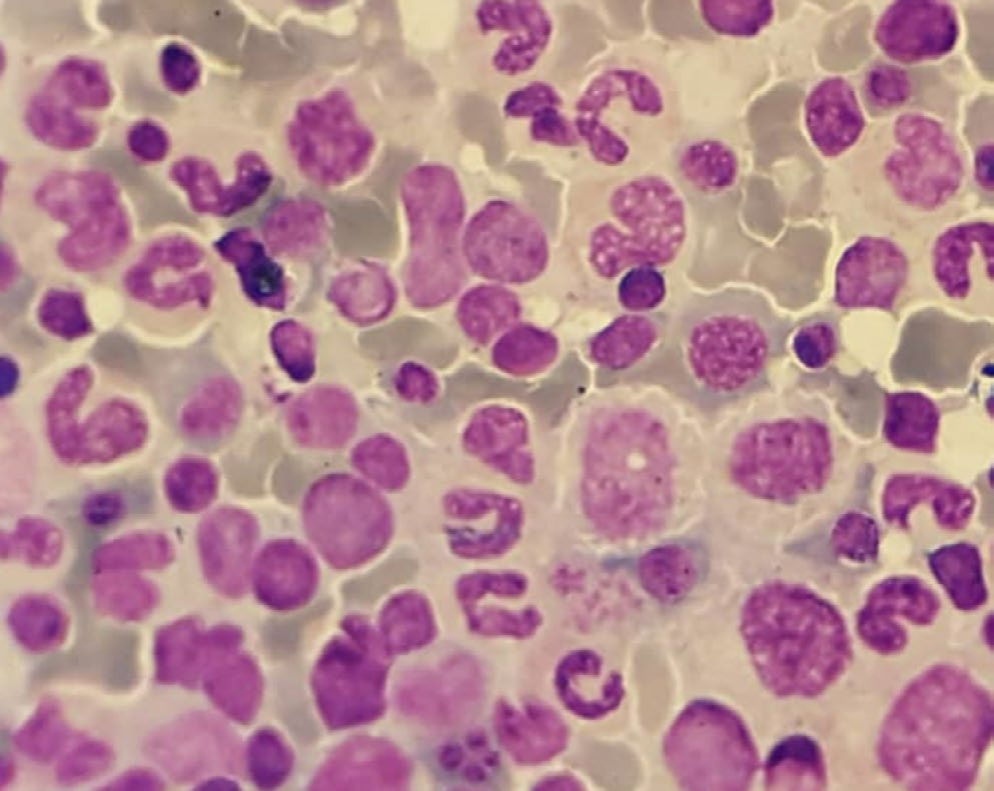
Bone marrow aspiration on day 30 of ATRA therapy in APL patient (100×). BM examination revealed the absence of promyelocytes with Auer rods which is a diagnostic criterion for complete remission of disease

On day 26 to day 32 of treatment, laboratory blood tests indicated a notable thrombocytosis with the platelet counts of 590 × 10^9^/L to 1280 × 10^9^/L (Figure 2). No known causes of thrombocytosis such as infections, hemorrhagic disorders, hemolytic anemia, and iron deficiency were observed.^9^ Peripheral blood smear revealed a notable thrombocytosis and slight anemia (Figure 3). Regarding the fact that the patient was asymptomatic, supportive care, ATRA, and ATO treatments were continued and ATRA dosage was not modified. Afterward, platelet number spontaneously started to decrease on day 32 of treatment so that its number was 400 × 10^9^/L on day 42 (Figure 2). On day 30 of ATRA therapy, BM examination showed a trilineal hematopoiesis with 1% of blasts and all criteria of morphological complete remission were observed (Figure 4). Four consolidation courses of treatments were planned as previously described.^10^, ^11^, ^12^, ^13^ After two consolidation courses of treatments, complete molecular remission was confirmed by the absence of PML‐RARA fusion transcript using RT‐PCR method.

## 3 DISCUSSION

As reported by previous study,^5^, ^14^ thrombocytosis is considered as a rare side effect of treatment of APL patients with ATRA. The result of our patient revealed that ATRA treatment combined with ATO induced bone marrow megakaryocyte differentiation and platelet production. Several mechanisms have been proposed to the regulation of platelet production.^15^ Thrombopoietin (TPO), a hormone usually produced by the liver and kidneys, is known as one of major mechanisms involved in the regulation of platelet production.^16^ TPO stimulates the differentiation, proliferation, and maturation of megakaryocyte, a cell precursor of platelet production.^17^ Another mechanism suggested to improve megakaryocytopoiesis is the release of immune agents such as IL‐1, tumor necrosis factor (TNF), IL‐2, IL‐3, IL‐11, IL‐12, IL‐6, and granulocyte macrophage‐colony stimulating factor (GM‐CSF),^14^ which are considered as inflammatory mediators.^18^, ^19^, ^20^, ^21^ During ATRA therapy, APL cell under differentiation can produce IL‐1β, IL‐6, IL‐8, and TNF‐α. IL‐1 and TNF‐α may participate in enhancement of platelet counts through inducing IL‐6 production.^22^ Although it is proposed the correlations of these factors, especially IL‐6, with the serum level of TPO,^15^ these associations have not well explained yet.

In a study on two APL patients who were treated with interferon alpha, Losada et al reported that platelet number was increased more than 1000 × 10^9^/L following treatment with ATRA.^14^ Thrombocytosis was not accompanied by other clinical complications.^14^ Subsequently, complete remission was obtained by ATRA therapy.^14^ Furthermore, another study on a 20‐year‐old man with APL revealed a thrombocytosis on day 29 of ATRA treatment. ATRA dose was not modified and the increased number of platelet started to reduce gradually on day 33 of treatment. Finally, the patient reached complete remission, without any complications associated with thrombocytosis.^15^ The results of our case were consistent with previous studies showing thrombocytosis during ATRA therapy.^11^, ^14^, ^15^ We observed an increased number of platelet (1280 × 10^9^/L) on day 32 of treatment. Thrombocytosis started to recover spontaneously on day 32 of ATRA, which is consistent with previous studies.^15^ Our data were agreed with other reports pointing complete remission without any complications correlated to thrombocytosis can be achieved following ATRA treatment.^12^, ^15^

## 4 CONCLUSION

These findings suggest that ATRA can induce severe thrombocytosis, as a potential side effect of treatment, in APL patients through stimulating the productions of different cytokines, especially IL‐6, from APL cells under differentiation. However, further studies and more information are needed to confirm this conclusion and provide criteria for its management.

## CONFLICT OF INTEREST

The authors report no conflict of interest.

## AUTHOR CONTRIBUTIONS

MH: carried out some of the experiments and collected the laboratory findings. RMA: participated in the design of the experiments. HM: drafted the manuscript and participated in the study design. All authors read and approved the final manuscript.

## ETHICAL APPROVAL

This study was approved by the Ethics Committee of Kashan University of Medical Science.

## Data Availability

All data generated or analyzed during this study are included in this published case report.
